# Ombitasvir/paritaprevir/ritonavir and dasabuvir±ribavirin for chronic HCV infection in US veterans with psychiatric disorders

**DOI:** 10.1002/jmv.25655

**Published:** 2020-02-17

**Authors:** Michael Fuchs, Alexander Monto, Norbert Bräu, Mariem Charafeddine, Warren Schmidt, Michael Kozal, Susanna Naggie, Ramsey Cheung, Gretja Schnell, Yao Yu, Kristine Richards, Victoria Mullally, Daniel E. Cohen, Doris Toro

**Affiliations:** ^1^ Hepatology Section, Hunter Holmes McGuire VA Medical Center Virginia Commonwealth University Richmond Virginia; ^2^ Department of Gastroenterology San Francisco VA Medical Center San Francisco California; ^3^ Viral Hepatitis Program James J Peters VA Medical Center New York City New York; ^4^ AbbVie Inc North Chicago Illinois; ^5^ Department of Internal Medicine Iowa City VA Healthcare System Iowa City Iowa; ^6^ Department of Internal Medicine, VA Connecticut Healthcare System, Yale School of Medicine Yale University New Haven Connecticut; ^7^ Department of Medicine Durham VA Medical Center Durham North Carolina; ^8^ Department of Gastroenterology and Hepatology VA Palo Alto Healthcare System Palo Alto California; ^9^ Gastroenterology Section VA Caribbean Healthcare System San Juan Puerto Rico

**Keywords:** comorbidity, direct‐acting antiviral, HCV GT1, substance‐related disorders, SVR, veterans

## Abstract

Hepatitis C virus (HCV) infections are more common among US veterans receiving care through Veterans Affairs (VA) Medical Centers than among the general population. Historically, HCV therapies had lower efficacy rates in VA patients, possibly due to common comorbidities such as psychiatric disorders and substance abuse. The direct‐acting antivirals ombitasvir/paritaprevir/ritonavir and dasabuvir (OBV/PTV/r+DSV)±ribavirin (RBV) are approved in the US for HCV genotype 1 (GT1)‐infected adults with or without cirrhosis. This study prospectively evaluated the safety and efficacy of OBV/PTV/r+DSV±RBV in VA patients with HCV GT1 infection. TOPAZ‐VA was a phase 3b, open‐label trial. Adult US veterans with HCV GT1 infection, without cirrhosis or with compensated cirrhosis, were eligible for enrollment. Patients with GT1a infection received OBV/PTV/r +DSV+RBV for 12 weeks or 24 weeks (for those with cirrhosis); GT1b‐infected patients without cirrhosis received OBV/PTV/r +DSV for 12 weeks; those with cirrhosis received OBV/PTV/r +DSV with RBV. The primary endpoint was sustained virologic response at posttreatment week 12 (SVR12); safety was also assessed. Ninety‐nine patients were enrolled at 10 sites from May through November 2015. The majority were male (96%), white (60%), and with GT1a infection (68%); 49% reported ongoing psychiatric disorders. Overall, 94% (93/99) achieved SVR12; three patients had a virologic failure. The most common AEs were fatigue (28%), headache (20%), and nausea (15%); six patients discontinued treatment due to AEs. In US veterans with HCV GT1 infection, OBV/PTV/r +DSV±RBV yielded a 94% overall SVR12 rate and was well tolerated. The presence of psychiatric disorders and/or injection drug use did not impact efficacy.

## Highlights

- Chronic hepatitis C virus (HCV) infection occurs at a higher prevalence in the VA Health System than in the general population.
- These patients are also at a higher risk of psychiatric disorders and substance abuse, which were historically risk factors for successful treatment of HCV.
- We treated 99 patients in the VA Health System with ombitasvir/paritaprevir/ritonavir and dasabuvir with or without ribavirin for 12 to 24 weeks.
- Sustained virologic response was achieved in 93 of 99 patients, with safety and tolerability comparable to the general population.
- The presence of psychiatric or substance abuse disorders did not impact treatment success.

## 1 INTRODUCTION

An estimated 1% of the US population, or approximately 3 million individuals, has chronic hepatitis C virus (HCV) infection.^1^ The prevalence of HCV infection among US military veterans is reported to be more than twice as high as in the general population and is approximately fourfold greater among US veterans when limited to the 1945 to 1965 birth cohort.^2^, ^3^ Interferon (IFN)‐based antiviral regimens demonstrated suboptimal efficacy and unfavorable safety profiles in US veterans who receive care within the Department of Veterans Affairs (VA).^4^, ^5^, ^6^ In addition, psychiatric comorbidities and substance use disorders are prevalent in VA patients, and IFN‐based HCV therapy is associated with psychiatric side effects, limiting its use in these patients.^7^, ^8^

The introduction of IFN‐free direct‐acting antivirals (DAAs) has provided additional treatment options for these patients. Approximately 80% of VA patients with HCV infection have HCV genotype 1 (GT1),^7^ and real‐world studies of the efficacy of DAAs in this population have demonstrated sustained virologic response (SVR) rates of 90% or greater in GT1‐infected patients.^6^, ^9^, ^10^ The 3‐DAA regimen of ombitasvir/paritaprevir/ritonavir (OBV/PTV/r) and dasabuvir (DSV)±ribavirin (RBV) demonstrated high SVR rates and a favorable safety profile in registrational trials in patients infected with HCV GT1^11^, ^12^, ^13^; however, these results have not been confirmed in the VA population. The TOPAZ‐VA study (NCT02442284) prospectively evaluated the efficacy and safety of OBV/PTV/r+DSV±RBV for 12 or 24 weeks in VA patients with GT1 infection, including those with ongoing psychiatric disorders and/or ongoing or a history of substance use.

## 2 METHODS

### 2.1 Study design and patients

The TOPAZ‐VA study was an open‐label, multicenter, phase 3b study conducted in 10 VA centers in the United States. Eligible patients were adult US military veterans with chronic HCV GT1 infection without cirrhosis or with compensated cirrhosis who were either treatment‐naive or had failed prior treatment with IFN, IFN/RBV, pegIFN/RBV, sofosbuvir and pegIFN/RBV, or sofosbuvir and RBV. Cirrhosis was determined based on previous liver biopsy, transient elastography, and/or serum biomarkers. Patients with active psychiatric conditions were eligible if the provider believed it was appropriate and they were able to provide informed consent and adhere to protocol requirements. Additional information on the determination of cirrhosis and eligibility criteria is in the Online Supporting Information.

### 2.2 Procedures

Patients received OBV/PTV/r (25/150/100 mg once daily) with DSV (250 mg twice daily) with or without weight‐based RBV (1000 mg for patients more than 75 kg or 1200 mg for patients ≥75 kg; divided twice daily) for 12 or 24 weeks. HCV GT1b‐infected patients received OBV/PTV/r+DSV for 12 weeks (without cirrhosis) or OBV/PTV/r+DSV+RBV for 12 weeks (with compensated cirrhosis); HCV GT1a‐infected patients received OBV/PTV/r+DSV+RBV for 12 weeks (without cirrhosis) or OBV/PTV/r+DSV+RBV for 24 weeks (compensated cirrhosis). HCV GT and subtype were determined from plasma samples using the Versant HCV Genotype Inno‐LiPA Assay, version 2.0 or higher (LiPA; Siemens Healthcare Diagnostics, Tarrytown, NY). HCV RNA levels were determined from plasma samples collected at all study visits and analyzed using the COBAS AmpliPrep/COBAS TaqMan HCV Test, v2.0, which has a lower limit of quantification of 15 IU/mL. Plasma samples were also collected for HCV resistance testing; details are in the Online Supporting Information.

### 2.3 Endpoints and statistical analyses

The primary efficacy endpoint of the study was SVR12 weeks after the last administered study drug dose (SVR12); the secondary efficacy endpoints were SVR12 in patients with active psychiatric conditions, the proportion of patients with on‐treatment virological failure, and the proportion of patients with posttreatment relapse by posttreatment week 12. Adherence was calculated as the percentage of tablets taken relative to total tablets expected to be taken; additional information is in the Online Supporting Information. Efficacy, safety, and demographic analyses were performed on the intent‐to‐treat (ITT) population, defined as all enrolled participants who received at least 1 dose of the study drug. Additional efficacy analyses were performed on a modified ITT population, which excluded patients who discontinued treatment for reasons other than virologic failure. The statistical review of the study was performed by a biomedical statistician at AbbVie.

### 2.4 Resistance analysis

For patients experiencing virologic failure, the amino acid sequences of HCV NS3/4A, NS5A, and NS5B from samples taken at baseline and at the time of virologic failure were determined by next‐generation sequencing. Resistance‐associated substitutions (RASs) were identified by comparison with the appropriate subtype‐specific reference amino acid sequence.

## 3 RESULTS

Of 115 patients screened, 99 were enrolled in the study and received study treatment. Demographics and baseline disease characteristics are shown in Table 1. The study population was 96% male and 40% non‐white race. Baseline FibroTest scores ranged from 0.18 to 1.0 (median: 0.66); aspartate aminotransferase to platelet ratio ranged from 0.18 to 8.1 (median: 0.56); and FIB‐4 score ranged from 0.60 to 12.24 (median: 1.90) (Table 1). Overall, 29% (29/99) of patients received the OBV/PTV/r+DSV for 12 weeks without RBV; the remaining patients received OBV/PTV/r+DSV+RBV for 12 weeks (55%; 54/99) or 24 weeks (16%; 16/99). Medications including prior HCV medication administered to patients before the treatment period and concomitant medications during the treatment period are listed in Table 2.

**Table 1:** Baseline characteristics

Characteristic	N = 99
Male, n (%)	95 (96)
Race, n (%)	
White	59 (60)
Black or African‐American	37 (38)
Other	3 (3)
Mean age (SD), years	61.5 ± 5.9
BMI ≥ 30 kg/m^2^, n (%)	30 (30)
HCV genotype, n (%)	
1a	67 (68)
1b	32 (32)
Treatment‐naive, n (%)	71 (72)
Treatment‐experienced, n (%)	28 (28)
HCV RNA ≥ 800 000 IU/mL, n (%)	81 (82)
FibroTest score, median (range), N = 98	0.66 (0.18‐1)
Albumin, median (range), g/L	43 (33‐51)
Platelet count, median (range), (×10^9^/L)	198 (64‐639)
APRI, median (range)	0.56 (0.18‐8.10)
Fibrosis‐4 score, median (range)	1.90 (0.6‐12.24)
Fibrosis stage, n (%)	
F0‐F1	43 (43)
F2	14 (14)
F3	22 (22)
F4	20 (20)
History of IDU, n (%)	59 (60)
Alcohol use, n (%)	
Current	21 (21)
Former	74 (75)
Never	4 (4)
Ongoing psychiatric disorder, n (%)	48 (49)
Type of psychiatric disorder, n (%)^a^	
Depression	36 (75)
Bipolar disorder	2 (4)
Schizophrenia	5 (10)
Anxiety and/or posttraumatic stress disorder	31 (65)

Abbreviations: APRI, aspartate aminotransferase to platelet ratio; BMI, body mass index; HCV, hepatitis C virus; IDU, injection drug use; SD, standard deviation.

^a^ Percent of each type of psychiatric disorder calculated as percent of patients with ongoing psychiatric disorder; patients can have more than one psychiatric disorder.

**Table 2:** Medications administered before and during the treatment period

Generic name, n (%)	N = 99
Any prior HCV medication	28 (28)
Medication used by ≥5% of patients	
Interferon	5 (5)
Peginterferon alfa‐2a	10 (10)
Peginterferon alfa‐2b	7 (7)
Ribavirin	25 (25)
Any prior medication	96 (97)
Medication used by ≥20% of patients	
Acetylsalicylic acid	21 (21)
Lisinopril	28 (28)
Omeprazole	29 (29)
Sildenafil	22 (22)
Any concomitant medication	96 (97)
Medication used by ≥20% of patients	
Acetylsalicylic acid	21 (21)
Lisinopril	28 (28)
Omeprazole	30 (30)
Sildenafil	22 (22)
Any concomitant psychiatric medication	46 (47)
Medication used by ≥10% of patients	
Bupropion	12 (12)
Mirtazapine	10 (10)

Abbreviation: HCV, hepatitis C virus.

**Figure 1:**
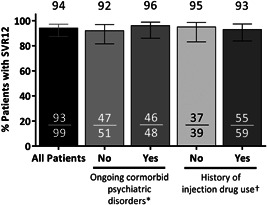
Efficacy. Rates of sustained virologic response (SVR) at posttreatment week 12 (SVR12) are shown in the overall intention‐to‐treat study population, and by ongoing psychiatric disorder status or history of drug use. *Comorbid psychiatric disorders include bipolar disorder, depression, schizophrenia, anxiety, posttraumatic stress disorder or other psychiatric disorder that are currently requiring pharmacotherapy. ^†^History of injection drug use was missing for one patient (patient achieved SVR12)

The SVR12 rate was 94% (93/99; 95% confidence interval [CI]: 87‐97%) in the ITT population (Figure 1). Of the six patients who did not achieve SVR12, one had an on‐treatment virologic failure at week 4 and two patients relapsed (one at posttreatment week 4 and one at posttreatment week 12). The remaining three patients failed due to premature discontinuation of study drug (one due to adverse events [AEs] and two due to withdrawal of consent). All of the patients who experienced virologic failure or relapse had a history of psychiatric disorder, including posttraumatic stress disorder, schizophrenia, bipolar disorder, and prior substance use. Of note, the patient who experienced a viral breakthrough on treatment was more than 80% compliant with the study drug. One patient who relapsed had baseline RASs in NS5A (Q30H and Y93H), but no baseline RASs in NS3 or NS5B. At the time of virologic failure, all three patients had RASs in NS3 and NS5A and one of three had RASs in NS5B (Table S1). One patient who achieved SVR12 had a late relapse between posttreatment weeks 12 and 24; details on this patient are included in Table S1. None of the three patients with virologic failure had cirrhosis or required an RBV dose modification. Among patients with ongoing psychiatric comorbidities, the SVR12 rate was 95.8% (46/48; 95% CI: 86.0‐98.8); the SVR12 rate was 93% (55/59; 95% CI: 83.8‐97.3) among patients with a history of injection drug use (Figure 1) Adherence to OBV/PTV/r was 99% (95/96), adherence to DSV was 98% (94/96), and adherence to RBV was 99% (67/68).

Treatment‐emergent AEs occurred in 80% (79/99) of patients. Fatigue, headache, nausea, insomnia, and pruritus each occurred in more than 10% of patients (Table 3). Ten treatment‐emergent serious AEs occurred in seven (7%) patients. One treatment‐emergent serious AE (mental status change due to drug interaction between DAAs and quetiapine) was considered possibly related to treatment. Six patients (6%) discontinued study drug due to a range of mild to moderate AEs, with no pattern of intolerance related to psychiatric disorders. One of these patients failed to achieve SVR12; this subject completed only 24 days of treatment and discontinued due to numerous complaints, both somatic (eg, diarrhea, dry skin, and asthenia) and neuropsychiatric (eg, dysesthesia, anger, anxiety, and paranoia), that were largely attributed to RBV. The other five patients who discontinued due to AEs completed between 57 and 114 days of study drug treatment and all achieved SVR12. Among patients receiving RBV, 19% (13/70) required RBV dose modification due to an AE, of which 62% (8/13) were due to a decrease in hemoglobin. No grade 3 or higher ALT elevations occurred during the study; one patient had a single grade 3 total bilirubin elevation, which was not associated with an AE.

**Table 3:** Key safety results

Adverse events (AEs)	N = 99
Any AE, n (%)	79 (80)
Serious AE^a^, n (%)	7 (7)
AE leading to discontinuation of study drug, n (%)	6 (6)^b^
AE leading to RBV dose modification, n/N (%)	13/70 (19)^c^
Deaths	0
AEs in ≥10% of patients, n (%)	
Fatigue	28 (28)
Headache	20 (20)
Nausea	15 (15)
Insomnia	10 (10)
Pruritus	10 (10)

Abbreviations: RBV, ribavirin, SVR, sustained virologic response.

^a^ Serious AEs were myocardial infarction, pancreatitis, cholangitis, and cholecystitis due to gallstones, viral gastroenteritis, appendicitis, full mouth teeth extraction due to periodontal disease, chronic obstructive pulmonary disease exacerbation, pneumonia, exacerbation of depressive symptoms, drug interaction causing altered mental state, and schizophrenia.

^b^ Five of six achieved SVR12.

^c^ No virologic failures among patients with RBV dose reduction.

## 4 DISCUSSION

In the TOPAZ‐VA study, HCV GT1‐infected VA patients treated with OBV/PTV/r+DSV±RBV for 12 or 24 weeks had an ITT SVR12 rate of 94%, consistent with findings of large real‐world observational studies of newer DAA regimens in US veterans. The treatment combination was well tolerated, and the safety profile was consistent with what has been previously reported in registrational trials.^12^, ^13^

During the IFN era of HCV treatment, psychiatric disorders and ongoing substance abuse were among the most common contraindications for HCV treatment among US veterans.^14^, ^15^ Here, an ongoing psychiatric disorder or a history of injection drug use did not impact SVR12 rates. These results are consistent with a real‐world analysis of 21 242 US veterans with HCV GT1 infection treated with DAAs, which found that a diagnosis of a mental health disorder or alcohol or substance abuse was not predictive of achieving virologic response.^10^ In another real‐world cohort of 1068 US veterans with HCV infection, neither psychiatric disorders nor substance abuse was predictive of virologic response, including in sub‐analyses of GT1 HCV‐infected patients or African‐American patients.^16^ Despite the high efficacy rates of IFN‐free DAAs, alcohol and drug abuse were still associated with low rates of DAA treatment initiation in US veterans.^17^ The high proportion of African‐American patients enrolled in this trial (38%) are a strength and allows the findings to be more readily generalized to the population of interest.

This study demonstrated high adherence (>98%) to all components of the OBV/PTV/r+DSV±RBV regimen, although assessment of adherence by pill count in clinical trials may be insensitive to dosing errors, for example, in the timing of administration. Two real‐world studies of US veterans used pharmacy records to assess adherence to treatment through medication procession ratio^5^, ^16^: individual adherence rates of 90% or greater were seen in 88% of patients treated with IFN‐based therapy^5^ and 94% of patients treated with an IFN‐free DAA regimen.^16^ In both studies, lower treatment compliance was associated with reduced SVR rates.^5^, ^16^

This prospective, interventional trial demonstrates the efficacy and safety of OBV/PTV/r+DSV±RBV in US veterans, including those with psychiatric or substance use disorders. While this regimen is no longer in use in the United States, the findings of this study are relevant to the use of other DAAs in this unique patient population, particularly with regard to the issue of potential drug‐drug interactions with psychiatric medications, which are likely to be of less concern with next‐generation therapies.^18^ A recent integrated analysis by Back et al^19^ confirms that patients with neuropsychiatric disorders can be safely and effectively treated with regimens such as glecaprevir/pibrentasvir.

In addition to the specific DAA regimen used, the limitations of this study include the small number of patients enrolled and the single‐arm, open‐label design. Patients with current heavy alcohol use were excluded from this study; therefore the impact of heavy alcohol use on virological response could not be assessed. A substantial proportion of the patients enrolled in this study had minimal fibrosis, and the majority were treatment‐naïve; thus, results may have differed if a greater proportion of patients had advanced fibrosis or were IFN‐experienced. While the small number of patients enrolled makes it difficult to conclude whether treatment responses differed by these baseline patient characteristics, previous registrational trials utilizing this regimen did not demonstrate a difference in efficacy between treatment‐experienced and treatment‐naïve populations,^13^, ^20^ or between patients with or without cirrhosis when treated according to the current product label.^12^

In conclusion, OBV/PTV/r+DSV±RBV was well tolerated in US veterans and achieved high SVR rates. In this population with a high rate of comorbidities, no new safety signals were observed; psychiatric and substance abuse disorders did not impact efficacy. These data confirm real‐world evidence supporting the use of IFN‐free DAA therapy for the treatment of HCV infection in US veterans with these comorbidities.

## CONFLICT OF INTERESTS

Michael Fuchs: research support to McGuire Research Institute: AbbVie, Gilead, Intercept, Conatus, Galectin, Genfit, BMS, Novartis; Royalties: Springer. Norbert Bräu: Research Support: AbbVie, Gilead, BMS; Speaker Bureau: AbbVie, Gilead, Merck; Advisory Boards: AbbVie, Gilead, BMS. Warren Schmidt: Investigator in AbbVie‐sponsored clinical studies. Michael Kozal: Yale/VACT receives research support from: Pfizer, Gilead, AbbVie, ViiV, and Bristol Myers Squibb for studies that Dr Kozal serves(d) as the primary investigator. Dr Kozal is an employee of the federal government and does not receive any salary support from these grants. Susanna Naggie: Research support: AbbVie, Gilead, BMS, Tacere. Ramsey Cheung: Investigator for AbbVie and Gilead. Mariem Charafeddine, Gretja Schnell, Yao Yu, Kristine Richards, Victoria Mullally, Daniel Cohen: Employees of AbbVie and may own stock or options. Alexander Monto: Investigator in AbbVie‐sponsored clinical studies. Doris Toro: Investigator in AbbVie‐sponsored clinical studies.

## AUTHOR CONTRIBUTIONS

MF, AM, NB, MC, WS, MK, SN, RC, GS, YY, KR, VM, DEC, and DT designed the study, performed the research, analyzed the data, provided feedback on manuscript preparation, and revised the manuscript for final submission.

## Supporting information

Supplementary information

## Data Availability

AbbVie is committed to responsible data sharing regarding the clinical trials we sponsor. This includes access to anonymized, individual, and trial‐level data (analysis datasets), as well as other information (eg, protocols and Clinical Study Reports), as long as the trials are not part of an ongoing or planned regulatory submission. This includes requests for clinical trial data for unlicensed products and indications. This clinical trial data can be requested by any qualified researchers who engage in rigorous, independent scientific research, and will be provided following the review and approval of a research proposal and the Statistical Analysis Plan and execution of a Data Sharing Agreement. Data requests can be submitted at any time and the data will be accessible for 12 months, with possible extensions considered. For more information on the process, or to submit a request, visit the following link: https://www.abbvie.com/our‐science/clinical‐trials/clinical‐trials‐data‐and‐information‐sharing/data‐and‐information‐ sharing‐with‐qualified‐researchers.html.
