# Supplementary material for: Ombitasvir/paritaprevir/ritonavir and dasabuvir±ribavirin for chronic HCV infection in US veterans with psychiatric disorders
Source: J Med Virol. 2020 Feb 17;92(12):3459–64. doi: 10.1002/jmv.25655 (PMC7687116; doi:10.1002/jmv.25655)
Supplement: Supplementary file 1 [file JMV-92-3459-s001.docx]

**SUPPORTING MATERIAL**

**Ombitasvir/Paritaprevir/Ritonavir and Dasabuvir ± Ribavirin for Chronic HCV Infection in US Veterans with Psychiatric Disorders**

Michael Fuchs^1^, Alexander Monto^2^, Norbert Bräu^3^, Mariem Charafeddine^4^, Warren Schmidt^5^, Michael Kozal^6^, Susanna Naggie^7^, Ramsey Cheung^8^, Gretja Schnell^4^, Yao Yu^4^, Kristine Richards^4^, Victoria Mullally^4^, Daniel E. Cohen^4^, Doris Toro^9^

Table of Contents

**Determination of Cirrhosis****3**

**Key Inclusion Criteria****3**

**Key Exclusion Criteria****4**

**Resistance Analyses****5**

**Adherence Monitoring****5**

**Table S1. Characteristics of Patients with Virologic Failure** **6**

**Determination of Cirrhosis**

Patients were considered to be noncirrhotic based on a liver biopsy within 24 months prior to or during screening that demonstrated the absence of cirrhosis (e.g., a METAVIR 49 Score of 3 or less, Ishak score of 4 or less) or, if a liver biopsy was unavailable, a screening FibroScan® result of < 12.5 kPa or a screening FibroTest score of ≤ 0.72 and aspartate aminotransferase (AST) to platelet ratio index (APRI) ≤ 2. 

Patients were considered to be cirrhotic based on previous histologic diagnosis of cirrhosis on a liver biopsy (e.g., METAVIR Score of > 3 [including 3 – 4 or 3/4], Ishak score of > 4) or, if a liver biopsy was unavailable, a screening or historic FibroScan score ≥ 14.6 kPa or a screening FibroTest > 0.72 and APRI > 2. 

In the absence of a qualifying liver biopsy, patients with a screening FibroScan result that was ≥ 12.5 kPa and < 14.6 kPa, a FibroTest result that was ≤ 0.72 and an APRI > 2, or a FibroTest result that was ≥ 0.73 and an APRI ≤ 2, were evaluated based on the investigator's clinical judgment to determine the presence or absence of cirrhosis.

**Key Inclusion Criteria**

1. US military veterans, male or female, currently receiving healthcare

through the Veterans Health Administration, and aged 18 years and older

2. Chronic HCV GT1 infection (plasma HCV RNA > 1000 IU/mL)

3. Treatment-naive or IFN, pegIFN, RBV, or sofosbuvir treatment experienced

4. No current or past clinical evidence of Child-Pugh B or C cirrhosis

5. No current heavy alcohol use (more than 5 drinks on the same

occasion on each of 5 or more days in the past 30 days)

6. Ongoing psychiatric disorders were allowed (defined as a clinical diagnosis of bipolar disorder, depression, schizophrenia, anxiety, post-traumatic stress disorder, or other psychiatric disorder and currently requiring pharmacotherapy)

**Key Exclusion Criteria**

1.          HCV genotype performed during screening that indicated infection with any genotype other than GT1.

3. Positive test result for HBsAg or confirmed positive anti-HIV Ab test.

4. Use of known strong and moderate inducers of cytochrome P450 3A (CYP3A) or any strong inducers of cytochrome P450 2C8 (CYP2C8) and strong inhibitors of CYP2C8 (e.g., gemfibrozil), as well as those that are contradicted for ritonavir and RBV, within 2 weeks or 10 half-lives of the medication, whichever was longer, prior to study drug administration.

5. Use of (or prior use of) any investigational or commercially available anti-HCV agents other than IFN, pegIFN, RBV, or SOF (including previous exposure to OBV, PTV, or DSV).

6. Clinically significant abnormalities or comorbidities that, in the opinion of the investigator, made the patient an unsuitable candidate for this study or its treatments. Patients with active psychiatric conditions (depression, bipolar disorder, schizophrenia, anxiety, and/or post-traumatic stress disorder) were eligible if they were able to provide informed consent and adhere to the protocol requirements, including attending all scheduled study visits.

7. Presence of heavy alcohol use (> 5 drinks on the same occasion on each of 5 or more days in the past 30 days).

8. Enrollment in another interventional clinical study or receipt of any investigational product within 6 weeks prior to study drug administration.

9. History of solid organ transplant, including liver transplantation.

10. Screening laboratory analyses that showed any of the following abnormal laboratory results:

● Calculated CrCl (by Cockcroft-Gault formula) < 30 mL/min

● Serum albumin < 2.8 g/dL

● International normalized ratio (INR) > 2.3. Patients with a known inherited blood disorder and INR > 2.3 could have been enrolled with permission of the AbbVie TA MD

● Hemoglobin < 11 g/dL for females or < 12 g/dL for males

● Total bilirubin > 3.0 mg/dL

● Platelet < 25,000 cells/mm^3^

11. Any clinical evidence (or any past clinical evidence) of Child-Pugh B or C Classification (Child-Pugh Score ≥ 7) or clinical history of liver decompensation such as ascites (noted on physical examination), variceal bleeding, or hepatic encephalopathy.

12. Confirmed presence of HCC indicated on imaging techniques such as computed tomography (CT) scan or magnetic resonance imaging (MRI) within 3 months prior to screening or on an ultrasound performed at screening for patients with cirrhosis (a positive ultrasound result was confirmed with CT scan or MRI).

**Resistance Analyses**

For patients with virologic failure, next generation sequencing at a 15% detection threshold was performed on plasma samples collected at baseline and the time of virologic failure. Signature amino acid positions included in the analysis were 155, 156, and 168 in NS3; 24, 28, 29, 30, 31, 32, 92, 93 in NS5A; 314, 316, 414, 446, 448, 451, 553, 554, 555, 556, 557, 558, 559, 561 in NS5B. The subtype-specific reference sequence for GT1a was 1a-H77 (GenBank Accession number NC_004102).

**Adherence Monitoring**

Drug compliance data (tablet counts) were recorded at each drug accountability visit and were used to assess compliance with study drug dosing. Compliance was calculated for each of the 3 tablet types as the percentage of tablets taken relative to the total tablets expected to be taken. Noncompliance with the study drug regimen was defined as a calculated percentage < 80% or > 120% for any of the following: OBV/PTV/r, DSV, and RBV. Taking more than the prescribed amount of tablets would results in values greater than 100%.

**Table S1. Characteristics of Patients with Virologic Failure***

|  | **Patient 1** | **Patient 2** | **Patient 3** |
| --- | --- | --- | --- |
| **HCV subtype** | GT1a | GT1a | GT1a |
| **Sex** | M | M | M |
| **Age** | 59 | 63 | 66 |
| **Fibrosis Stage** | F3 | F3 | F0-F1 |
| **Treatment-experience** | Naïve | Experienced | Naïve |
| **IL28B** | CC | TT | CT |
| **Psychiatric history†** | Depression/ Bipolar disorder | Schizophrenia | PTSD |
| **Alcohol use** | Former | Former | Former |
| **IDU** | Former | Former | No |
| **Time of VF** | PTW12 | PTW4 | TW4 |
| **NS3 substitutions**^**‡**^ |  |  |  |
| **At baseline** | None | None | None |
| **At VF** | D168H | D168Y | D168V |
| **NS5A substitutions**^**‡**^ |  |  |  |
| **At baseline** | Q30H, Y93H | None | None |
| **At VF** | Q30H, Y93H | M28T | M28T |
| **NS5B substitutions**^**‡**^ |  |  |  |
| **At baseline** | None | None | None |
| **At VF** | None | None | S556G |
| HCV, hepatitis C virus; GT, genotype; PTSD, post-traumatic stress disorder; IDU, injection drug use; VF, virologic failure; PTW, post-treatment week; TW, treatment week. ^*^One 57 year old male with HCV GT1a infection and F2 fibrosis relapsed between 12 and 24 weeks post-treatment; possibility of reinfection was ruled out by phylogenetic analysis. This patient had baseline RASs Q30H and Y93H in NS5A, and at time of failure had Q30H+Y93H in NS5A and S556G in NS5B. ^†^All 3 patients had history of asymptomatic conditions (no treatment). ^‡^ Next-generation sequencing data based on a 15% detection threshold.  Positions included in the analysis were 155, 156, and 168 in NS3; 24, 28, 29, 30, 31, 32, 92, 93 in NS5A; 314, 316, 414, 446, 448, 451, 553, 554, 555, 556, 557, 558, 559, 561 in NS5B. |  |  |  |
